# SEOM clinical guidelines for the prophylaxis of infectious diseases in cancer patients (2021)

**DOI:** 10.1007/s12094-022-02800-3

**Published:** 2022-03-01

**Authors:** Isabel Echavarria, J. Rafael Carrión Galindo, Jesús Corral, María Pilar Diz Taín, Fernando Henao Carrasco, Vega Iranzo González-Cruz, Xabier Mielgo-Rubio, Teresa Quintanar, Carlos Rivas Corredor, Pedro Pérez Segura

**Affiliations:** 1 Department of Medical Oncology, Hospital General Universitario Gregorio Marañón, Instituto de Investigación Sanitaria Gregorio Marañon (IiSGM), CIBERONC, C/ Dr. Esquerdo, 46, 28007 Madrid, Spain; 2 Deparment of Medical Oncology, Hospital Universitario del Sureste, Madrid, Spain; 3 Department of Medical Oncology, Clínica Universidad de Navarra, Madrid, Spain; 4 Department of Medical Oncology, Hospital Universitario de León, León, Spain; 5 Department of Medical Oncology, Hospital Universitario Virgen Macarena, Sevilla, Spain; 6 Department of Medical Oncology, Hospital General Universitario de Valencia, Valencia, Spain; 7 Department of Medical Oncology, Hospital Universitario Fundación Alcorcón, Madrid, Spain; 8 Department of Medical Oncology, Hospital General Universitario de Elche, Elche, Spain; 9 Department of Medical Oncology, Hospital de Mataró, Barcelona, Spain; 10 Department of Medical Oncology, Hospital Clínico San Carlos, Madrid, Spain; 11 Department of Medicine, Universitat de València, Valencia, Spain; 12 Centro de Investigación Biomédica en Red en Cáncer (CIBERONC), Valencia, Spain

**Keywords:** Vaccination, Prophylaxis, Immunosuppression, Chemotherapy

## Abstract

Infections are still a major cause of morbi-mortality in patients with cancer. Some of these infections are preventable through specific measures, such as vaccination or prophylaxis. This guideline aims to summarize the evidence and recommendations for the prevention of infections in cancer patients, devoting special attention to the most prevalent preventable infectious disease. All the evidences will be graded according to The Infectious Diseases Society of America grading system.

## Introduction

Cancer incidence is steadily increasing, and while anticancer therapies are evolving, infections are still responsible for significant morbi-mortality in patients with cancer, owing to a greater risk of infection and a certain degree of immunosuppression [1, 2].

### Immunosuppression in patients with cancer

Immunity compromise in patients with cancer is multifactorial, and it is driven by patient comorbidities, tumor characteristics and anticancer treatments. Advanced tumors are associated with a dysfunction of systemic immunity partly due to tumor progression itself. For instance, in patients with advanced non-small cell lung cancer, systemic immunosuppression has been characterized by an increased number of myeloid-derived suppressor cells (MDSCs), regulatory T lymphocytes, and immunosuppressive cytokines [3], and in patients with colorectal carcinoma, a systemic inflammatory response that promotes an immunosuppressive environment has also been described [4]. Other factors related to advanced solid tumors, such as malnutrition, comorbidities, smoking, systemic inflammation and obstruction of anatomical structures, also contribute to a higher risk of infection [2]. In addition, disruption of natural defensive barriers, as in the case of mucositis, is an important promoter of infections in patients with cancer on active treatment.

On the other hand, anticancer therapy plays a key role in the development of immunosuppression in cancer patients. Myelotoxicity, and especially neutropenia, are one of the main toxicities of classical chemotherapeutic agents. Febrile neutropenia, defined as fever > 38 °C and neutropenia < 500 cells/ml, is a potentially dangerous complication with risk of sepsis. The incidence of febrile neutropenia with certain chemotherapeutic regimens has prompted the use of granulocyte colony growth stimulating factors (G-CSF) as primary or secondary prophylaxis, as well as antimicrobial prophylaxis in specific settings [5].

Novel therapeutic agents such as immune checkpoint inhibitors do not carry a significant risk of myelotoxicity, but may require prolonged treatment with high-dose corticosteroids for the treatment of the immune-related adverse events (irAE). This prolonged use of high-dose glucocorticoids, either for the treatment of irAE or because of central nervous system metastases or other tumor complications, is also associated with an immunosuppressive state, mediated by their interference with key inflammatory signaling responses such as depression of the phagocytic function of macrophages and alveolar neutrophils, suppression of dendritic cell maturation and function, and restriction of inflammatory cell migration in areas of infection, which facilitates the development of pneumonia [6].

### Morbidity and mortality of infections in cancer patients

Infections in cancer patients lead to a significant increase in morbidity and mortality, with a risk of mortality from sepsis up to 10 times higher than patients without cancer [7, 8]. Therefore, the detection of active and latent infections prior to chemotherapy, antimicrobial prophylaxis guidelines, vaccinations, and early detection and treatment of infections are especially important.

## Methods

This Spanish Society of Medical Oncology (SEOM) Guideline has been drawn up by 10 oncologists members of SEOM. The Infectious Diseases Society of America grading system has been used to classify the level of certainty and grade of recommendation [9] (Table 1).

**Table 1:** The Infectious Diseases Society of America grading system

Levels of evidence	
I	Evidence from at least one large randomized, controlled trial of good methodological quality (low potential for bias) or meta-analyses of well-conducted randomized trials without heterogeneity
II	Small randomized trials or large randomized trials with a suspicion of bias (lower methodological quality) or meta-analyses of such trials or of trials with demonstrated heterogeneity
III	Prospective cohort studies
IV	Retrospective cohort studies or case–control studies
V	Studies without control group, case reports, expert opinions
Grades of recommendation	
A	Strong evidence for efficacy with a substantial clinical benefit, strongly recommended
B	Strong or moderate evidence for efficacy but with a limited clinical benefit, generally recommended
C	Insufficient evidence for efficacy or benefit does not outweigh the risk or the disadvantages (adverse events, costs, etc.), optional
D	Moderate evidence against efficacy or for adverse outcome, generally not recommended
E	Strong evidence against efficacy or for adverse outcome, never recommended

Infectious Diseases Society of America grading system. Adapted from [9]

### Infection control practices for adult patients with solid tumors

#### General recommendations

Due to the high morbi-mortality related to infections in cancer patients, hygienic measures represent the initial step toward infection prevention, and should be maximized. Although evidence of their clinical benefit is lacking, these standard precautions are endorsed by expert panels [10, 11], since benefits outweigh harms [12] (III-A):

- hand hygiene and use of personal protective equipment whenever risk of exposure exists (gloves, masks and gowns).
- Respiratory hygiene and cough etiquette.
- Injection safety and venous catheter care.
- Cleaning and disinfection of devices and environmental surfaces.
- Transmission precautions.
- Dietary recommendations, avoiding potentially contaminated food.
- Immunizations, following recommendations by the CDC/IDSA [13].

### Vaccination in patients with cancer

#### Types of vaccines [13]

Vaccination is a cornerstone of infection prevention, and is especially relevant in cancer and other immunocompromised patients.

Different types of vaccines exist:

- Live-attenuated vaccine: based on weakened versions of the pathogen, they may carry a risk of virus replication and infection in immunocompromised hosts.
- Inactivated vaccine: inactivated vaccines, made from inactivated or killed pathogens, do not carry a risk of replication and infection, but may generate lower immune responses and therefore require booster doses.
- Subunit/conjugate vaccine: made from specific proteins or carbohydrates from the germ, they do not carry a risk of replication and infection, but booster shots may be needed.
- mRNA vaccines: made from viral RNA, they do not entail a risk of infection.

Immunogenicity of vaccines in patients with cancer may be hampered due to immunosuppression. Data on vaccine efficacy among patients with cancer are scarce, since most data come from underpowered studies that included patients with a variety of cancers and chemotherapeutic regimens, and that used diverse definitions of vaccine response, mainly by assessing seroconversion [14, 15]. Moreover, vaccine efficacy can be very difficult to assess in the oncological population owing to a low incidence of the disease or seasonality.

#### Timing of immunizations [13, 15]

In addition to the scarce data on vaccine efficacy in patients with cancer, timing of immunization must be taken into account in this population:

- Vaccines should preferably be administered to cancer patients before the start of the treatments (systemic therapies, radiation or splenectomy); at least 2 weeks before in the case of inactivated vaccines (I-A) and 4 weeks for live-virus vaccines (I-A), when feasible.
- Live-virus vaccines should not be given to patients with cancer receiving chemotherapy or other immunosuppressive therapies due to the risk of vaccine-derived infections (II-D). Inactivated vaccines can be safely administered during chemotherapy, but may be ineffective, and thus, they should not be considered valid doses unless seroconversion is documented (II-A).
- After completion of chemotherapy, patients who have not received anti-B cell antibodies can receive both inactivated and live-virus vaccines 3 months after the end of the treatment if clinically indicated (III-A). Patients treated with anti-B cell antibodies should wait at least six months for vaccination (III-B).

Vaccines should be given before the start of systemic therapies when feasible (I-A). Live-virus vaccines are contraindicated during chemotherapy (II-D) (Table 2).

**Table 2:** Summary of recommendations

		Level of evidence
Vaccination	Vaccines should be given before the start of systemic therapies when feasible	I-A
	Live-attenuated vaccines must not be administered during chemotherapy treatment	II-D
	SARS-CoV-2 vaccination is recommended in patients with cancer	V-C
	Influenza vaccine should routinely be administered in patients undergoing cancer treatments	I-A
	Patients with cancer should receive the PCV13 vaccine followed by the PPSV23	II-A
	Vaccination against Herpes zoster should be administered to patients with cancer on active treatment	II-A
Viral hepatitis prophylaxis	Screening for HBV and HCV should be performed before the start of anticancer treatments	I-A
	Patients with chronic HBV (HbsAg-positive) undergoing anticancer treatment should receive antiviral prophylaxis during treatment and for at least 12 months after its completion	I-A
	Patients with past HBV and solid tumors with a low-risk of reactivation, may be closely monitored	IV-B
Antimicrobial prophylaxis	Antibiotic and antifungal prophylaxis for the prevention of febrile neutropenia is not routinely recommended in patients with solid tumors	II-A
	PJP prophylaxis, with TMP-SMX as the preferred regimen, is recommended in patients with prolonged high-dose corticosteroids treatments	III-A

#### SARS-CoV-2 vaccination

The global pandemic of severe acute respiratory syndrome coronavirus 2 (SARS-COV-2) has impacted significantly patients with cancer. Regular contact with health-care system has led to an increased exposure and risk of infection, and cancer-related immunosuppression has placed them at a higher risk of developing severe COVID-19 and death (5–61%) than the overall population (2–3%) [16–18]. Among patients with cancer, those with hematological and lung malignancies, advanced stage and ongoing antineoplastic treatments seem to be associated with an increased risk of mortality. However, it is important to note that the higher incidence and severity of COVID-19 in cancer patients is based on retrospective and non-comparative studies, and many unmeasured confounding factors and selection biases might exist (IV-C). On the other hand, SARS-CoV-2 infection could have a negative impact on the screening, diagnosis, treatment and follow-up in patients with cancer, leading to an increased morbidity and mortality due to their tumors (V-C).

Initially, despite data on safety and efficacy of SARS-CoV-2 vaccination in cancer patients were limited, vaccination was highly recommended in this setting (V-C). However, there is growing evidence that vaccination in this population is safe and that patients with solid tumors have seroconversion rates similar to the general population, although seroconversion may take longer to achieve [19, 20]. In fact, recent data on the efficacy of SARS-CoV-2 vaccination in patients with solid tumors have shown that the majority of the patients develop antibodies against the virus (anti-S1), as well as cellular response following the infection and vaccination, regardless of the tumor type or treatment. Hematological malignancies are associated with lower rates of seroconversion, especially among patients treated with B cell-depleting agents. However, seroconversion may overestimate the level of immunity against the infection, and especially, against variants of concern, since a significant proportion of cancer patients do not reach a neutralizing antibody titer. Importantly, immunity is much higher following infection in patients with prior infection, supporting the administration of a 3rd dose in cancer patients [21, 22]. There is no consensus about the optimal timing of vaccination for patients on active treatment. Healthcare staff vaccination is strongly recommended to reduce nosocomial transmission of the disease to cancer patients [23] (III-C).

Anti-SARS-CoV-2 vaccination is highly recommended in patients with cancer (V-C).

#### Influenza vaccine in cancer patients

Seasonal influenza virus infections are an important cause of respiratory disease and cancer patients are at an increased risk of morbi-mortality compared to the general population [24]. Two main types of influenza virus, A and B, are responsible for the majority of the cases of severe disease, and mutations in their surface antigens prompt the annual epidemics [25]. Influenza virus-related mortality can reach up to 9–10% in cancer patients undergoing active therapy. This can be prevented trough influenza vaccination, since it has shown a reduction in the duration and severity of the infection, and a significant decrease in the influenza-associated morbidity and mortality [26].

The efficacy and safety of influenza vaccination in cancer patients receiving conventional chemotherapy have been evaluated in several studies, showing a decreased rate of seroconversion compared with immunocompetent patients [27, 28]. Among patients treated with checkpoint inhibitors, influenza vaccination seems to safe and effective, with adequate seroconversion rates observed in the studies available [29]. There are controversial data on the effect of the timing of vaccination during chemotherapy cycles on the immunogenicity of the vaccine, and thus, there is no clear recommendation about it [25, 27].

Since influenza vaccination in cancer patients in active treatment has shown to be safe and effective, and contributes to the reduction of morbi-mortality, inactivated viral vaccines against influenza should be administered annually before the start of the season (I-A). Vaccination rates among cancer patients, their relatives, and healthcare staff still need to be improved, and quality data on the optimal timing of vaccination during the chemotherapy cycle is highly needed.

#### Pneumococcal vaccine

Patients undergoing chemotherapy for solid tumors are at a 40–50% higher risk for the development of invasive pneumococcal disease (IPD) than the general population, with fatality rates approaching 35% [30]. Two pneumococcal vaccines, the pneumococcal 13-valent conjugated vaccine (PCV13) and the pneumococcal 23-valent polysaccharide vaccine (PPSV23), are currently available, and studies have suggested that vaccination reduces the burden of IPD and non-bacteremic pneumococcal pneumonia.

In 2013, the Advisory Committee on Immunization Practices (ACIP) and the Centers for Disease Control and Prevention recommended the pneumococcal vaccination of unvaccinated, immunocompromised patients aged 19 years or older, based on the administration of PCV13 followed by the PPSV23 within 6 to 12 weeks (II-A) [31].

For patients who have previously received PPSV23, the PCV13 dose should be given at least 1 year after the last PPSV23 dose. For those who require additional doses of PPSV23, the first such dose should be given no sooner than 8 weeks after the PCV13 dose [32].

Pneumococcal vaccination is strongly recommended in patients with cancer on chemotherapy treatment (II-A).

#### Herpes zoster vaccine

Patients with solid tumors are at an increased risk of developing herpes zoster, a reactivation of the varicella zoster virus. A recombinant zoster vaccine has shown significant efficacy in the prevention of herpes zoster and post-herpetic neuralgia. Clinical trials carried out in patients with solid tumors have shown strong immune responses following vaccination [33]. Although vaccine shortage has prevented from routine vaccination, it will presumably be overcome soon.

When available, recombinant zoster vaccine should be administered to patients with cancer undergoing chemotherapy, with a schedule of two doses spaced two months apart (II-A).

#### Other routine vaccines

All cancer patients should be up to date on tetanus and diphtheria toxoid (Td) immunization, although limited information is available regarding Td vaccine response in this setting. Its administration is recommended at least 10 days before initiating chemotherapy (II-A) [34].

Patients with cancer should follow the same ACIP recommendations for meningococcal vaccine as compared with other immunosuppressed patients. It should be offered either prior to chemotherapy or once the patient’s immune system has recovered (III-C) [15].

Vaccination against Hepatitis B (HBV) and Hepatitis A (HAV) should be considered in patients with incomplete vaccination status or indicated refresher vaccination. Data showed immunogenicity and safety in patients under chemotherapy. In addition, in Germany, the Standing Committee on Vaccination (STIKO) recommends vaccination against HAV for patients receiving transfusions (II-B) [35].

Live-attenuated vaccines, such as measles, mumps, and rubella (MMR) (II-B) and varicella (II-A), are contraindicated in highly immunocompromised cancer patients. They could be administered at least 4 weeks before the initiation of chemotherapy and at least 3 months after completion. It is advisable to vaccinate susceptible household contacts of cancer patients to minimize the likelihood of their infection.

Recent studies have highlighted the fact that international travel is common among patients living with cancer, even immunocompromised patients [36]. Live-attenuated yellow fever vaccine, oral typhoid vaccine, and oral cholera vaccine cannot be given to them (II-D).

### Vaccination in patients undergoing splenectomy

Some particular situations, such as patients undergoing a splenectomy in the context of their cancer surgery, may require specific prophylaxis. These patients are at increased risk of infection by encapsulated bacteria and other pathogens, and therefore, should follow a specific vaccination schedule, including vaccination against *N. meningitidis*, in addition to the influenza and pneumococcal vaccines, preferably at least 2 weeks before the splenectomy or 2 weeks after (III-A) [37]. Special attention to fever and infectious symptoms must be paid, and early antibiotic initiation must be considered.

#### Human papillomavirus vaccine (HPV)

In addition, some infectious agents are associated with the development of malignancies, such as HPV, related to anogenital (including cervical, vaginal, vulvar, and anal) and oropharyngeal cancers. Infection by high-risk HPV genotypes, such as 16 and 18, is responsible for approximately 70% of cervical cancers. The ACIP and the Centers for Disease Control and Prevention recommend routine administration of the recombinant 3-dose HPV vaccine to females and males aged 11 or 12 years old. For those not vaccinated at the target age, vaccination is recommended up to age 26 years (II-A). Immunosuppression is not a contraindication for HPV vaccination [38]. In HPV-positive women, vaccination may prevent from other HPV types and reduce the risk of relapse, and therefore, should be considered [39] (I-A).

## Antiviral prophylaxis

### Hepatitis screening for patients with cancer prior to therapy

Cancer patients with HBV and hepatitis C virus (HCV) infection receiving systemic therapy are at risk of reactivation. Among patients with solid tumors, HBV reactivation is estimated around 25% and 3% for chronic HBV and past HBV infection respectively, and may reach up to 48% and 18% respectively among those with hematological malignancies. Likewise, immune checkpoint inhibitors also carry a significant risk of HBV reactivation, which may be up to 21%, with the additional risks of autoimmune hepatitis and further immunosuppression due to frequent glucocorticoids treatment. HBV reactivation is associated with a variety of liver disease, ranging from transient asymptomatic elevation of transaminases to fulminant hepatitis and death [9, 40, 41]. Risk of reactivation for each specific anticancer agent has not been established. Therefore, serological status before therapy should be assessed (I-A), although it should not delay the start of the oncological systemic therapy [9, 40–45].

HCV reactivation studies in cancer patients are not as numerous as for HBV, and the prevalence of HCV infection in cancer patients ranges from 1.5% to 32% [46]. However, an increased mortality in cancer patients with HCV infections compared to HCV-negative patients has been noticed, due to a higher risk of early cirrhosis and viral reactivation [45, 47].

### Management of HBV reactivation in cancer patients

*Patients with chronic HBV (HbsAg-positive):* antiviral prophylaxis therapy should be started preferably 2 weeks before the start of immunosuppressive therapies, and continued for the duration of cancer therapy and through the 12 months following the last cycle of cancer treatment. Monitoring includes serum HBV DNA levels and ALT at baseline and every 6 months [9, 40–42] *(I-A)*. An exception may be patients receiving hormonal therapy alone, since the risk of reactivation is low. Counseling of their infectious risk to others is recommended.

*Patients with prior HBV infection (HbsAg-negative/anti-HBc-positive)* and systemic therapy not associated with a high risk of reactivation, such as conventional chemotherapeutic agents used in solid tumors, should be carefully monitored, including DNA levels, HbsAg and ALT, so potential reactivations can be detected and antiviral therapy started (IV-B). This is due to the belief that reactivation of past HBV infections only occurs with strong immunosuppression. On the other side, patients treated with cancer therapies with high risk of HBV reactivation, such as anti-CD20 monoclonal antibodies or hematopoietic stem cell transplantation, should receive antiviral prophylaxis for at least 12–18 months after completion of treatment, with individualized management thereafter *(I-A) *[9, 40–42, 48].

#### Prophylaxis, treatment and vaccination

In patients not vaccinated and with no prior exposure to HBV (HbsAg, anti-HBc and anti-HBs negative), risk of infection exists and therefore, vaccination is recommended, preferably at least 3 to 6 months after cessation of treatment [46]. Serological testing following vaccination may be considered to ensure immunity.

Treatment with entecavir or tenofovir during anticancer therapy is recommended for the prophylaxis or treatment of HBV reactivation *(II-A)* [49]. HIV should be ruled out before starting these drugs. Preferably, antivirals should be started at least two weeks before immunosuppressive treatment, especially in patients with detectable levels of HBV DNA; and continued for a minimum of 1 year after cessation of immunosuppressive treatment *(I-B)*, although anticancer therapy initiation should not be delayed [9, 40–42, 48].

### Treatment of hepatitis C in cancer patients.

Serologic testing for HCV should be assessed in all candidates for immunosuppressive therapy *(I-A)* [45], and subsequently, HCV-RNA viral load should be measured in serologically positive patients *(III-A).* Patients with HCV-RNA detectable, and thus, with current HCV infection, should receive further diagnostic assessment of the extent of liver disease. Treatment is recommended for all patients with acute or chronic HCV infection *(I-A)*, except for those with a short-life expectancy because of non-liver-related comorbidities *(II-B)* [43]. The preferred simplified regimens with HCV direct-acting antiviral (DAA) drug combinations are: glecaprevir/pibrentasvir for a duration of 8 weeks or sofosbuvir/velpatasvir for a duration of 12 weeks *(I-A)*. All these treatments aim to achieve a sustained virologic response, with undetectable HCV-RNA levels in serum or plasma. These treatments should not be given concomitantly with anticancer treatments.

## Antimicrobial prophylaxis during neutropenia

Intensive cytotoxic chemotherapy can cause febrile neutropenia (FN) episodes and life-threatening infections. The risk of infection increases with the depth and duration of neutropenia (< 500 cells/mm^3^ for > 7 days), so that patients are stratified as high-risk (> 7 days) and low-risk (≤ 7 days) according to its anticipated duration [12, 50, 51]. Prophylactic antibacterial, antifungal, and antiviral agents can reduce infectious complications, especially among high-risk patients, such as patients with acute myeloid leukemia/myelodysplastic syndromes (AML/MDS) or undergoing HSCT. On the other hand, most chemotherapy regimens used for solid tumors are associated with short-duration neutropenia episodes (≤ 7 days), and therefore, prophylaxis is not recommended (II-D) [12, 50–52]. Risk of FN and the need for antimicrobial prophylaxis should be carefully assessed.

Antibacterial prophylaxis has shown to be effective and safe in preventing FN and bacterial infections, with a significant reduction of infection-related mortality and rate of bacteremia [53]. Prophylaxis with fluoroquinolones (FQ), such as ciprofloxacin and levofloxacin, should be considered in the high-risk patients detailed above (I-B) [12, 50, 51, 53, 54]. Combination of FQs with agents active against Gram-positive infections is generally not recommend (II-D). Antibiotic prophylaxis is not routinely recommended for solid cancer patients (II-D).

Antifungal prophylaxis. Among patients at high risk of invasive fungal infection (IFI), mostly related to *Aspergillus* spp. and *Candida* spp., oral triazoles, such as posaconazole (I-A) and voriconazole (II-B), are the preferred choice, although other options like echinocandins are available [12, 50, 52, 54]. Patient and treatment singularities may affect the antifungal preference. Antifungal prophylaxis is not routinely recommended for patients with solid cancers (II-D).

Antiviral prophylaxis. Patients seropositive for herpes simplex virus undergoing allogeneic HSCT or induction chemotherapy for acute leukemia should receive antiviral prophylaxis with acyclovir or valacyclovir (II-A). HSCT recipients who are seropositive for varicella zoster virus (VZV) should receive antiviral prophylaxis, preferably with valacyclovir (II-A) [12, 50, 54]. For autologous HSCT, prophylaxis for HSV/VZV is also recommended (II-A) [55]. Cytomegalovirus reactivation does not generally occur in patients with chemotherapy-induced neutropenia. Serologic testing for HSV and VZV, and antiviral prophylaxis, is not indicated in solid tumor patients (II-D).

Duration of antimicrobial prophylaxis. Prophylaxis is recommended during the expected period of neutropenia, and until mucositis resolution, whichever occurs later, in the case of antiviral prophylaxis. VZV antiviral prophylaxis should be continued for at least 1 year [12, 50, 51, 54].

## Pneumocystis prophylaxis in prolonged corticosteroid treatments

*Pneumocystis jirovecii* pneumonia (PJP) is becoming an increasingly matter in HIV-negative patients, and carries a poorer prognosis than in the HIV-positive patients. Several risk factors have been identified in HIV-negative patients, being the prolonged use of high-dose corticosteroids treatment (≥ 20 mg/day prednisone equivalent for 4 weeks) the most common predisposing risk factor in cancer patients, although hematological malignancies patients are also at a greater risk of infection [56]. These patients are at high risk of PJP, regardless of the underlying type or stage of malignancy, or use of other chemotherapeutic agents, either during high-dose therapy or the steroid-tapering period. In this setting, prophylaxis is strongly recommended (III-A), with TMP-SMX as first-choice agents (II-A) [12, 51, 57], either a single-strength (80/400 mg) tablet daily or a double-strength tablet (160/800 mg) daily or three times per week (II-B) [51, 57]. Prophylaxis should be continued while steroids are being weaned and/or for a period of 6 weeks after cessation. Although the CD4 count has not been shown to correlate with risk of PCP in patients HIV-negative, some experts have suggested CD4 monitoring as a method to quantify risk of disease development and to guide duration of prophylaxis, taking as threshold 200 cells/mm^3^ (V-C) [57]. Thus, PJP prophylaxis is recommended, with TMP-SMX as the preferred regimen, for patients with a prolonged use of high-dose corticosteroids (III-A).

## Conclusion

In conclusion, infections are still a major issue in patients with cancer, but some of them are preventable trough vaccination or prophylaxis, and thus, these measures represent a cornerstone of cancer patients’ management.
